# Magnetic sense-dependent probabilistic decision-making in humans

**DOI:** 10.3389/fnins.2025.1497021

**Published:** 2025-03-07

**Authors:** In-Taek Oh, Soo-Chan Kim, Yongkuk Kim, Yong-Hwan Kim, Kwon-Seok Chae

**Affiliations:** ^1^Brain Science and Engineering Institute, Kyungpook National University, Daegu, Republic of Korea; ^2^Department of Electrical and Electronic Engineering, Research Center for Applied Human Sciences, Hankyong National University, Anseong, Republic of Korea; ^3^Department of Mathematics, Kyungpook National University, Daegu, Republic of Korea; ^4^Neuroscience Program, School of Allied Health Sciences, Boise State University, Boise, ID, United States; ^5^Department of Biology Education, Kyungpook National University, Daegu, Republic of Korea

**Keywords:** decision-making, probability, magnetic sense, humans, binary choice, geomagnetic field, magnetoreception, magnetic field resonance

## Abstract

Even though it is not well characterized how much humans can sense the geomagnetic field (GMF), numerous magnetosensitive animals can detect GMF broadly as a sensory cue, when a spatial decision-making is needed for orientation or migration. In an article of recent series of studies, we showed that the empirical probabilities of stone selections in Go game were significantly different from the theoretical probability. In this study, we assessed the implication of the GMF in modulating subconscious non-spatial decision-making in human subjects and the underlying mechanism with exploiting the zero-sum binary stone selection of Go game as a proof-of-principle. In a laboratory setting, the experimental probability in a decision-making was significantly hampered by the cancelation of the ambient GMF. Moreover, the attenuation of decision-making was confirmed by a specific range of magnetic resonance radiofrequency. In numerous stone selection games among amateur Go players in the artificial magnetic field setting, the analyses of stone selection rate by trials and steps for decision-making pinpointed the subconscious stone selection as a primary modulating target in the binary decision-making. Our findings may provide unique insights into the impact of sensing GMF in probabilistic decision-making in which theoretical probability is manifested into empirical probability through a magnetic field resonance-dependent mechanism.

## Introduction

There is a mount of supporting evidence that the magnetic field on Earth (geomagnetic field, GMF) plays an important role as a sensory cue for a long- or short-distance migration (Johnsen and Lohmann, 2005; Wiltschko and Wiltschko, 2006; Lohmann et al., 2012; Guerra et al., 2014; Bae et al., 2016; Oh et al., 2020), a body alignment (Begall et al., 2008; Hart et al., 2013; Bazalova et al., 2016), food foraging (Bae et al., 2016; Oh et al., 2020), and magnetic imprinting (Oh et al., 2020) in numerous animal species. Studies on human magnetoreception of the GMF are relatively rare, and the results that humans can sense the GMF have been controversial (Baker, 1980; Westby and Partridge, 1986; Mulligan and Persinger, 2012; Wang et al., 2019; Shibata et al., 2024). However, two recent studies demonstrated that human males can sense the GMF by a blue light (Schulten et al., 1978; Ritz et al., 2000; Chae et al., 2022) via the magnetic field resonance-dependent mechanism (Chae et al., 2019; Chae et al., 2022), wherein the external Larmor frequency magnetic field (the frequency of the oscillatory magnetic moment of single electrons in the radical pairs by the Zeeman interaction) affected certain spin reactions in the light-activated magnetoreception (Hore and Mouritsen, 2016). These results are consistent with the core concept of radical pair mechanism, which explains how a magnetic field can influence reaction kinetics by affecting electron spin dynamics (Wiltschko and Wiltschko, 2006; Hore and Mouritsen, 2016). Indeed, humans also express a putative magnetoreceptor cryptochrome protein in the eyes (Thompson et al., 2003) which might act based on a radical pair mechanism (Schulten et al., 1978; Ritz et al., 2000; Hore and Mouritsen, 2016), similarly as the GMF-sensing migratory birds (Hochstoeger et al., 2020; Xu J. et al., 2021).

The GMF-influenced orientations toward four cardinal (Chae et al., 2019)- or two alternative (Chae et al., 2022)-magnetic directions in humans were mediated through an inclination (Chae et al., 2019) or non-canonical inclination compass (Chae et al., 2022) only under the low blood glucose level derived from short-term fasting (~ 20 h) (Chae et al., 2019; Chae et al., 2022). Interestingly, fasted men but not women were magnetosensitive under the food-associative (Chae et al., 2019; Chae et al., 2022) or non-associative (Chae et al., 2022) behavioral paradigms, and magnetic sensitivity appeared to be activated in a low range of blood glucose (Chae et al., 2019; Chae et al., 2022). These results suggest that fasting is a feasible prerequisite for a study on human magnetic sensation. However, the impact of GMF on decision-making has been barely reported, although directional guide and cognitive implications have been suggested in humans and other animals (Sarimov et al., 2008; Zhang et al., 2021). For examples, the impairments of cognitive function including locomotor reactions and memory recollection in humans (Sarimov et al., 2008) and adult hippocampal neurogenesis in mice (Zhang et al., 2021) were reported under the hypo-GMFs.

Animals constantly face environmental changes to adopt the most favorable option for enhancing the odds of their survival (Stevens and Ruxton, 2018). Likewise, humans can make near-optimal decisions in two alternative forced-choice tasks with insufficient information using largely unknown strategies (Bogacz et al., 2006; Kilpatrick et al., 2019). Recent studies showed that humans use mental navigation in conceptual spaces to search for relative or absolute directions (Viganò et al., 2021a), and novel symbolic words matching to audio-visual stimuli can be represented on mono- or bi-dimensional cognitive maps in the brain (Viganò et al., 2021b). Thus, we postulated that the input on two-alternative directions through GMF sensing in humans (Chae et al., 2022) could evolutionarily have been incorporated into an abstract decision-making framework with the cognitive maps as a reference, when ordinary sensory modalities such as the five senses are not effective. In the context, if the input on one direction corresponds to one of the two choices, then the other input on the opposite direction may be inclined to push forward the other choice. Although individual factors may not enable to determine either direction, numerous factors combined may contribute to push forward to one direction, not the other as a whole (Vargas and Lauwereyns, 2021).

Here, our main hypothesis is that magnetic directional information from the GMF sensing can impact our abstract decision-making in binary choices. To test this hypothesis, we adopted the zero-sum stone selection of Go games (Chae et al., 2023) to investigate the potential implication of GMF for inducing a discrepancy between the empirical and theoretical probability. Contrary to the common expectations, the study supported that actual chance for human binary choice may not be 50:50, in the empirical data from professional Go matches and stone selection games between amateur Go players. Hence, our results suggest that the stone selection in Go games can be used as a model paradigm to resolve the debate regarding the discrepancy between theoretical and empirical probability by elucidating the underlying mechanism. In this study, we assessed the role of GMF and the possible mechanism of influencing our binary decision-making in the artificially generated magnetic field. We focused on assessing 3 paradigms to show which factors potentially contribute to generate the discrepancy between theoretical and empirical probabilities in our experimental settings as below: (1) Stone selection games in the laboratory under the near-zero intensity of GMF showed the correlation between “Black stone (%)” and different visual cues and/or fasting duration, (2) Trial-based and stepwise analyses of the data from the paradigm 1 showed the pattern of lower “Black stone (%)” at near-zero in “odd” of white stones, (3) Under the different radiofrequency magnetic fields, a resonance magnetic field (Larmor frequency) influenced the “Black stone (%).”

## Methods

### Subjects

Participants are composed of 55 men (age, 21–27 years; mean, 23 years; body mass index, 20–34 kg/m^2^; mean, 24 kg/m^2^) and 53 women (19–26 years, mean 21 years; body mass index, 15–30 kg/m^2^, mean 21 kg/m^2^) volunteers without reported physical disabilities or mental disorders including color blindness and claustrophobia (Chae et al., 2019; Chae et al., 2022). All the subjects were Korean undergraduate students at Kyungpook National University who are not familiar with the rules of Go games at all or have limited knowledge, especially for the stone selection process, and thus they were not categorized by the level (Dan). They were informed of the objective of the study, experimental procedures, and financial compensation for participation and were asked to follow the rules for the study. Nevertheless, the specific intentions or experimental designs including “control” or “placebo” were blind to the subjects. To motivate the subjects to win the game and achieve a higher black stone (%), two kinds of financial rewards were provided individually — one for winning a game and the other for higher monthly black stone (%). Before each experiment, subjects underwent short-term fast for either ~7 h (around 9:00 am–4:00 pm or 11:00 am–6:00 pm) or ~ 20 h (around 2:00 pm–10:00 am or 6:00 pm–2:00 pm), depending on the fast duration and time point for experiments. Neither food nor medical treatments except pure water was allowed during the fast between the last meal and test (Chae et al., 2019; Chae et al., 2022). To exclude the insufficient sleep effect, normal night sleep that is at least 6 h between 10 pm and 8 am on the test day, was required (Chae et al., 2019; Chae et al., 2022). Before starting each experiment, the subjects were stabilized on a chair for approximately 15 min in a waiting room next to the testing room. In particular, the subjects were supervised to remove any colored lens and detachable metallic or electromagnetic items including coins, watches, glasses, earrings, hairpins and mobile phones from their bodies. Based on the assessments of a pre-experiment questionnaires and blood glucose levels on the predetermined subjects before starting the first game (see ‘Stone selection experiments’ below), any subjects who had not followed these rules were rescheduled. Some subjects were excluded from several experiments for personal reasons, such as conflicting schedule or COVID-19-related symptoms. The study was approved in advance by the Institutional Review Board of Kyungpook National University (KNU-2021-0153). All experiments were performed in accordance with relevant guidelines and regulations for human subject research, and informed consent was obtained from all the subjects.

### GMF modulation and magnetic fields oscillation

**Figure 1:**
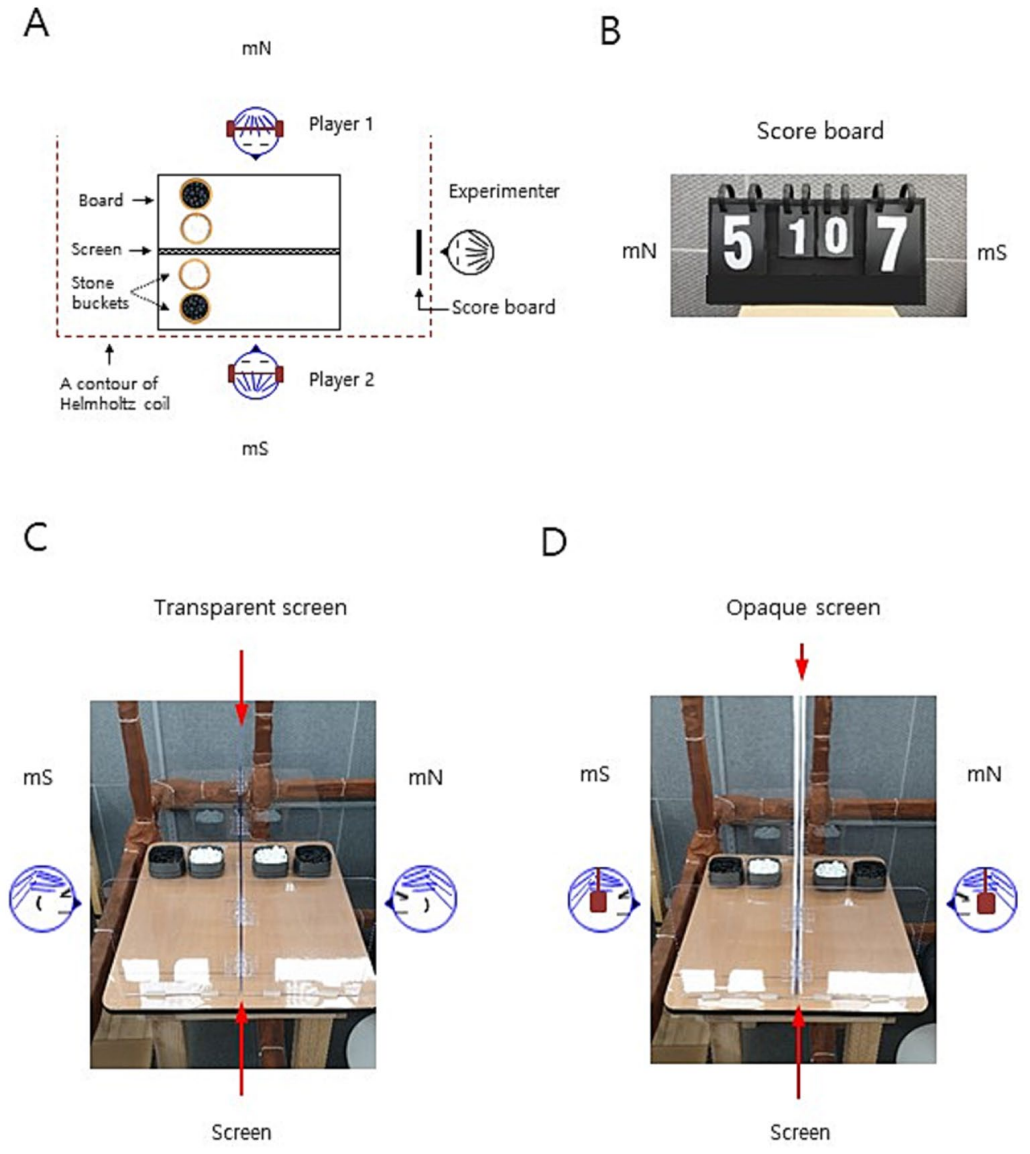
The experimental setup for the stone selection paradigm. **(A)** A schematic drawing of the experimental setup for the stone selection paradigm. A top view of two players facing each other across the board with an experimenter who sat east side from the players to conduct experiments. Two players swapped the seat before the second set of a game by the same experimental procedure. A transparent or opaque screen stood on the midline of the board between the players; the latter was combined with the ear-muffing of the players. A scoreboard was maintained by an experimenter, so that real-time score was noticed by the players. mN and mS, the ambient magnetic north and south direction, respectively; rectangular solid line, the contour of the board; red dashed line, the outline of the symmetric lower half of Helmholtz coils for the vertical axis and the upper half was omitted for simplicity; black and white circles, two sets of stone buckets on the board for each player at both seats. **(B)** A scoreboard was maintained to post a real-time set and game scores to the players by an experimenter. Large numbers indicate the score in a set, while small numbers are game scores for the north and south seat players at a certain time point; mN and mS, the north and south seat direction, respectively. In this example, the game score is “1 to 0” and the real time score of the ongoing set in the second game is “5 to 7” for the north and south seat player, respectively. **(C,D)** An experimenter’s views of the transparent or opaque screen on the board between the players are displayed. mN and mS, the ambient magnetic north and south direction, respectively.

The ambient GMF in the core of the Helmholtz coils in a testing room was applied with the total intensity of 45.0 μT, inclination of 53, and declination of −8 (Daegu City, Republic of Korea), which were maintained in the laboratory throughout the period of stone selection experiments. The testing room was shielded by a rectangular parallel-piped Faraday cage comprising 10 mm thick aluminum plates, and grounded during the entire experiment (Chae et al., 2019; Chae et al., 2022). To provide the subjects’ eyes with the indicated GMF-like magnetic fields, the same coil system from our previous studies (Bae et al., 2016; Chae et al., 2019; Oh et al., 2020; Chae et al., 2022) was applied to modulate the total intensity of the magnetic field, depending on the experimental conditions (Supplementary Table S1). It comprised three double-wrapped, orthogonal, and rectangular Helmholtz coils (1.89 × 1.89 m, 1.89 × 1.80 m, and 1.98 × 1.98 m for the north–south, east–west, and vertical axes, respectively) electrically-grounded with copper mesh shielding. A player sat on a non-metallic homemade chair at the center of the Helmholtz coils (north seat) with his head positioned in the middle space of the vertical axis of the coils, and the other player sat on the same type of chair outside of the coils (south seat) on the ambient magnetic north–south axis (Figures 1A,C,D). The two players sat facing each other at the distance of 1.30 m across the board of 60 × 70 × 72 cm (L × W × H), and an experimenter sat on a non-metallic chair outside of the coils at the distance of 1.35 m east from the two players to conduct the experiments. The modulated GMF at the glabella of subjects on the north seat varied markedly as indicated, but the accompanying change in the GMF at the same region of subjects on the south seat was relatively marginal (Supplementary Table S1). The field homogeneity at the position of the subject’s head was approximately 95%, as measured using a 3-axis magnetometer (MGM 3AXIS; ALPHALAB, USA) (Chae et al., 2019; Chae et al., 2022). The oscillating magnetic fields, RF 1 and RF 2 (1.260 and 1.890 MHz, respectively; the mean intensity 100 nT for each) were exposed vertically (37° relative to the ambient GMF) to the north seat players during the corresponding set of the game (approximately 13 min) (Chae et al., 2022). To produce the oscillating magnetic fields, the same system comprising a function generator, amplifier and calibrated coil antenna, was used as reported in the previous study (Chae et al., 2022). The measurements of oscillating magnetic fields were performed on the glabella of the subjects using the same spectrum analyzer with the calibrated loop antenna and magnetometer (the band widths of the RF 1 and RF 2 were 0.020 and 0.019 MHz (“average,” √3 kHz), respectively, at the bottoms of the peaks), as in the previous study. The electromagnetic noise in the cage including the switch button module for GMF modulation and the antenna for generating oscillating magnetic fields was measured and maintained constantly (see ref. Chae et al., 2022). The temperature at the position of the subjects’ head was maintained at 25 ± 0.5°C (Data logger 98,581; MIC Meter Industrial, Taiwan) (Chae et al., 2019; Chae et al., 2022).

### Stone selection experiments

To habituate the subjects to the stone selection game, all the subjects participated in two preliminary games between two randomly matched opponents by the same procedures of the ‘*in situ* stone selection games’ which followed the same rule in professional Go matches (see Kim et al., 2025) with little to no differences, under the supervision of the experimenters in the waiting room. As described in our recent report (Kim et al., 2025), the stone selection procedures are following: Both players sat face-to-face across the board as in Figure 1A. The game comprised two sets and 20 trials of stone selection per set. (1) Both players took a turn to grab white stones in the trials in a set—player 1 grabbed white stones in the first trial, and player 2 grabbed white stones in the second trial for the stone selection (see Supplementary Data Sheet S1 for record form and an example). Participants were instructed to grab at least 10 white stones in the step 1. In case of grabbing less than 10 at the step 1, the trial was nullified and retried. A participant was instructed not to use a predetermined pattern but it was spontaneously decided whether the number of black stones would be one or two in the step 2 during the entire game. The game information was recorded on a report form for each trial, set, and game by the participants themselves, with confirmation of both players under the supervision of an experimenter. The correction rate of judgment error in the game information was approximately 2%. (2) Before starting the second set of a game, the player 1 swapped the seat with player 2. The same game rules and procedures in 1 above were applied to the second set. (3) The black stone (%) of a player in a game was calculated as the number of black stone selection out of total number of trials (e.g., 18/40 × 100 = 45%). (4) All the participants played the second game with randomly arranged another player within a group. The preliminary game results were not included in the final data analysis.

The stone selection experiments in the testing room were conducted according to the same rules in the preliminary games with some differences. (1) Experiments were performed within 90 min window at 10:00–11:30 am, 2:00–3:30 pm, 4:00–5:30 pm or 6:00–7:30 pm (local time, UTC + 09:00) (time spent for an experiment: 50–70 min; mean ≈ 1 h). The subjects underwent different durations of fast depending on the experiment, as described above. If the determined blood glucose levels of any of the two players before the first game varied by more than 15% relative to the mean (Chae et al., 2022), the experiment was postponed to a later date (approximately 2% of experiments). (2) In the experiment, two randomly predetermined subjects belonging to a subject group of the same sex (men or women) were tested in two consecutive games under different experimental conditions. The two subjects were randomly assigned as a player 1 or 2 in the first game by an experimenter and then swapped the position in the second game. Before the experiments, the subjects were asked to sit facing toward the opponent player throughout the games, while they were allowed to turn their head or eyes toward the experimenter to see hand cues for the game progress or the scoreboard and any directions to refresh their mind for decision-making. Nonetheless, they were instructed not to pay attention to the experimental sets other than the experimenters’ guides, stone selection/counting, and scoreboard. Either the transparent acrylic or opaque double-sided hardboard acrylic screen (0.3 × 60 × 50 cm and 2 × 60 × 90 cm, respectively; L × W × H; Figures 1C,D) stood on the board between the players, depending on the games. In the ‘opaque screen’ games, both players had earplugs and earmuffs to prevent auditory cues. Subjects at both seats were illuminated by light from a diffused light-emitting diode (Chae et al., 2022) during games with a transparent or opaque screen (290 or 280 lux (lx) on the glabella, respectively). Before the first game in the experiment, both players were reminded of the two kinds of financial rewards as follows: the financial compensation for the participation, and the additional reward for the selection of black stone. (3) The same procedure as 1 in the ‘preliminary stone selection games’ above was conducted by an experimenter’s strict step-by-step hand cues (see Supplementary Video S1). As shown in the movie, the size of stone receptacles (9.3 × 9.3 cm) was large enough for subjects to conveniently grab white stones as they wanted. The experimenter collected and recorded the game information for each trial and set on the record form (Supplementary Data Sheet S1 for record form and an example). A non-metallic scoreboard (Figure 1B) stood in front of an experimenter and was manually maintained by the experimenter, so that a real-time score could be provided to the players. The experimenter confirmed the correctness of the written information on the record form at the end of the set. (4) The players switched the seats for the second set of games under the guidance of an experimenter, and the same procedure in 3 was carried out in the second set. (5) After the second set of information was validated, the black stone (%) for each player in a game was calculated as the number of black stone selection out of total number of trials (i.e., 40) × 100 (A correction rate for error in a game is approximately 1%). The game score was posted on a scoreboard for players to see. (6) According to the procedures 3–5 above, the two players participated in the second game under different experimental conditions by the supervision of an experimenter. After the completion of the experiment, the subjects were asked to separate in the waiting room and then the post-experiment questionnaire was conducted based on the recorded game information. The questions cover what they experienced during the experiment including whether they could perceive magnetic fields or experience any strange feelings at any moments during the game and intentionally grab white stones to be odd or even in the step 1. Experiments under different conditions were dispersed and performed in a random order with an interval of at least 3 days for the same subject between experiments. All experiments were performed in a double-blinded manner. The experimenters who conducted the stone selection games were aware of the experimental conditions including the fast duration of the subject, the type of screen (transparent or opaque), and whether the subject was wearing filtered or non-filtered goggles. However, the experimenters were not aware of the purpose of each experiment without knowing the intents of the different experimental conditions. Another experimenter who analyzed the data, was not aware of the experimental conditions. Thus, none of the experimenters was fully aware of all the information including the subjects, experimental conditions, collected data, and process of data analysis.

### Statistical analysis

To determine the significance of data, a two-sample *t*-test or the percentile bootstrap method (Chae et al., 2022) was applied using the software Origin 2019 (OriginLab, Northampton, USA). The analysis of the *north* seat players’ data from the stone selection experiments in the laboratory was performed as below. The black stone (%) for the north seat player in a game was calculated as the number of black stone selection out of total number of trials at the north seat (i.e., 20) × 100. For the trial-based analysis, the black stone (%) of all subjects were averaged for each trial in the 1–20th trials. The detailed calculation formulas for the trial-based and stepwise analyses are described in Supplementary Data Sheet S2. To verify the suitability of the *t*-test, each of the group datasets was examined using the Anderson-Darling test to determine if the data showed a normal distribution (Chae et al., 2022) (Supplementary Data Sheet S3). To determine if the difference between the means of the two data sets was significant, the two-sample *t*-test was used when the two data sets followed a normal distribution (Supplementary Data Sheet S4), and the percentile bootstrap method (Efron, 1982; Chae et al., 2022) was employed (95% confidence interval) if any of them deviated from a normal distribution (Supplementary Data Sheets S5–S9 for raw data). To evaluate the blood glucose level, the two-sample *t*-test or percentile bootstrap method was adopted depending on the results of the Anderson-Darling test, as described above. To address the issue of multiple comparisons and to mitigate the likelihood of type I errors, appropriate adjustments were applied to the *p*-values obtained from each method. For the *t*-tests, the Benjamini-Hochberg procedure was applied to the *p*-values derived from the *t*-tests to reduce the false-positive discovery rate. The critical value for each *p*-value was calculated as (i/m) *k*, where ‘*i*’ is the rank of the *p*-value in ascending order, ‘*m*’ is the total number of comparisons, and ‘*k*’ is the significance level. Results were considered significant if the *p*-value meets that ‘*P* is less than the critical value’ (Supplementary Data Sheet S4) (Benjamini and Hochberg, 1995). For the bootstrap tests, a bootstrap-based minimum *p*-value adjustment was implemented to account for family-wise error rate. During each bootstrap iteration, *p*-values for all tests were computed, and the smallest *p*-value was recorded. After multiple iterations (re-samplings), the distribution of minimum *p*-values was constructed. Adjusted *p*-values for the observed data were computed based on their position in this distribution, ensuring robust error control across all the bootstrap tests (Supplementary Data Sheet S5) (Davison and Hinkley, 1997). These adjustments ensured the validity and reliability of the statistical inferences while addressing the challenges associated with multiple testing. Statistical values are presented as the mean ± standard error of the mean (SEM). n.s., not significant by a two-sample *t*-test; N.S., not significant by the percentile bootstrap analysis (Efron, 1982; Chae et al., 2022). *p*-value *, < 0.05 or **, < 0.01 by a two-sample *t*-test; #, < 0.025 (or > 0.975) by the percentile bootstrap analysis, were regarded as significant.

## Results

### The geomagnetic field influences the empirical probability in probabilistic abstract decision-making

**Figure 2:**
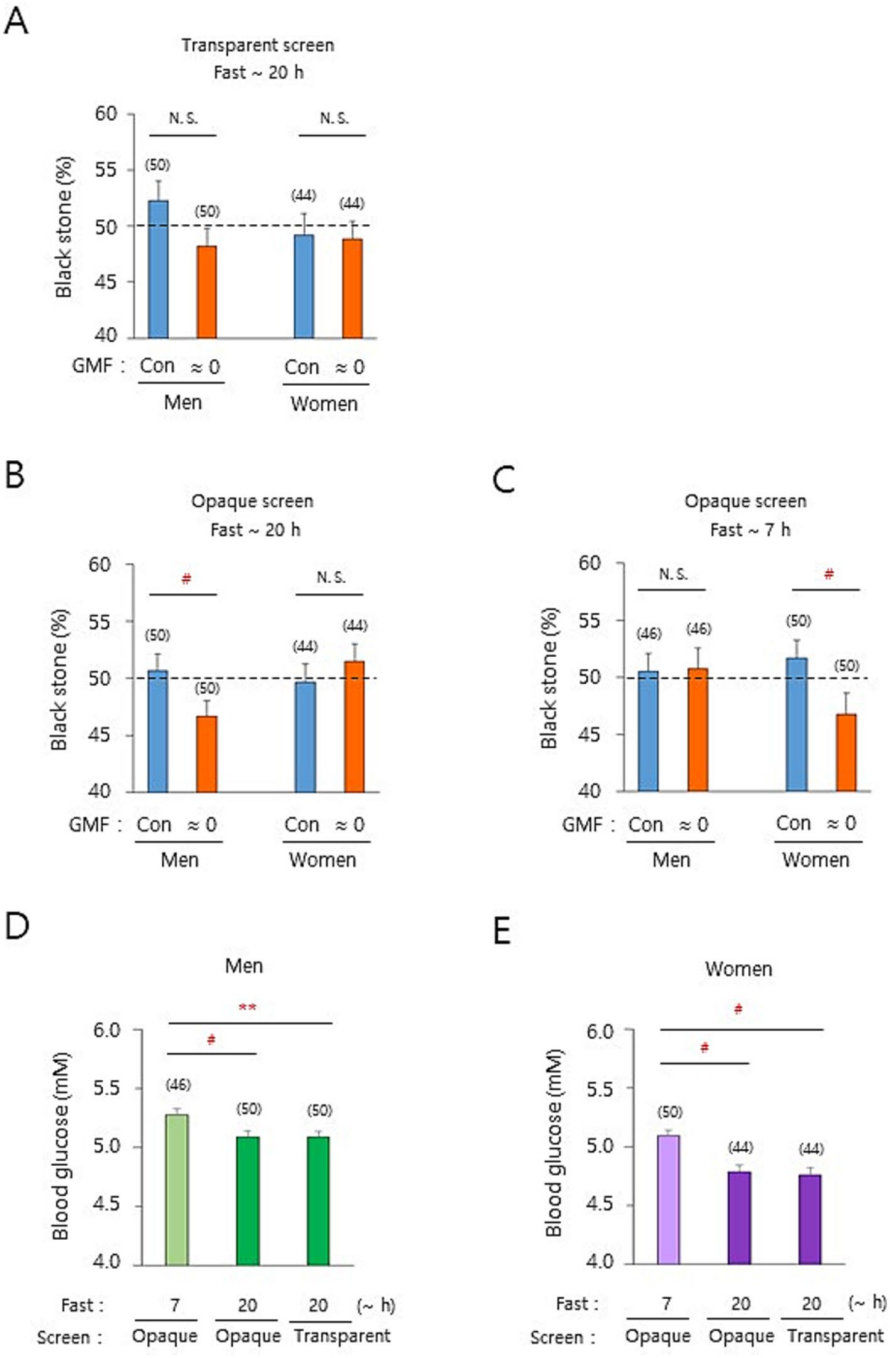
The geomagnetic field influences the empirical probability of probabilistic abstract decision-making. **(A–C)** Black stone (%) under different experimental conditions are indicated above each of the graphs. Note the significant differences in the black stone (%) in men **(B)** and women **(C)**. **(D,E)** Blood glucose levels in different fast conditions were determined shortly before the first game of a stone selection experiment. GMF, geomagnetic field; Con, control (the ambient GMF); ≈ 0, near-zero GMF; Statistical values, mean ± standard error of the mean (SEM); N.S., not significant and ^#^, *p* < 0.025, by the percentile bootstrap analysis; **, *p* < 0.01 by a two-sample *t-*test; horizontal dashed lines, the theoretical probability (50%) for black stone (%). The number of subjects in each group is indicated above bars in graphs.

Due to the intriguing observations (Kim et al., 2025), we have investigated the underlying mechanism of the discrepancy between theoretical and empirical probabilities by exploiting the stone selection paradigm as a proof-of-principle. Since it is unknowable who will be a winner and how visual/auditory cues or surroundings impact their decisions despite both players are equally engaged in the stone selection process, we reasoned that the player 1 subconsciously grab white stones to be odd or even and/or the player 2 can pick 1 or 2 black stones to increase his/her winning chances in the stone selection by implicitly using certain aspects of GMF. In order to test whether this postulation was meaningful in the setting of modulated GMF, a player’s head was situated to locate around the core of the Helmholtz coils (north seat) in a three-dimensional space, and the other player sat on a chair outside of the coils (south seat) of the magnetic north–south axis (Figure 1A; Paradigm 1 above, see Methods). Note that the ambient GMF at the north seat but not at the south seat was markedly modulated by the Helmholtz coils, depending on the experimental conditions (Supplementary Table S1). The players were allowed to see the score board freely throughout the experiments (Figures 1A,B). All the data analyses were based on the black stone (%) for north seat players in the zero-sum game paradigm. Therefore, both players were provided with even experimental conditions by randomization and taking a turn for fair games. In addition, based on our previous findings that short-term fasting is a prerequisite for men’s geomagnetic sensations (Chae et al., 2019; Chae et al., 2022), we assessed the fasting effects on potentially differential sex responses with fasting male and female subjects for up to 20 h. Then, we performed the stone selection (20 trials between the same players at each seat/game) with a transparent screen set first between the players (Figure 1C and Supplementary Video S1). The black stone (%) for the north seat players was appreciably decreased for men by the cancelation of GMF to near-zero intensity (near-zero GMF), compared to that under the ambient GMF, whereas it was not different for women (Figure 2A and Supplementary Data Sheet S10). In contrast, the near-zero GMF significantly reduced the rate for men but not for women with an *opaque* screen combined with ear-muffing (Figure 1D) to minimize any potential visual or auditory effects by the opponent or surroundings (Figure 2B and Supplementary Data Sheet S10). The near-zero GMF additionally induced a significant decrease in the rate for women but not for men under the same opaque screen condition (Figure 2C and Supplementary Data Sheet S10) after a short fast (~ 7 h), in which fasting duration was not assessed in our previous studies (Chae et al., 2019; Chae et al., 2022). Interestingly, the same glucose level in the blood (mean value, 5.1 mol/L) under different fasting conditions for men (~ 20 h, Figure 2D and Supplementary Data Sheet S10; *t*(94) = 2.68, *p* < 0.01) and women (~ 7 h, Figure 2E and Supplementary Data Sheet S10) suggests a potential causal relationship between the glucose level and reduced black stone (%) under the near-zero GMF (Figures 2B,C). Taken together, empirical probabilities in the binary choice were significantly affected by the GMF and food context for men and women differently, suggesting that the GMF plays a role in affecting binary decision-making in a particular range of blood glucose in humans.

### Geomagnetic sensing may affect decision-making subconsciously

**Figure 3:**
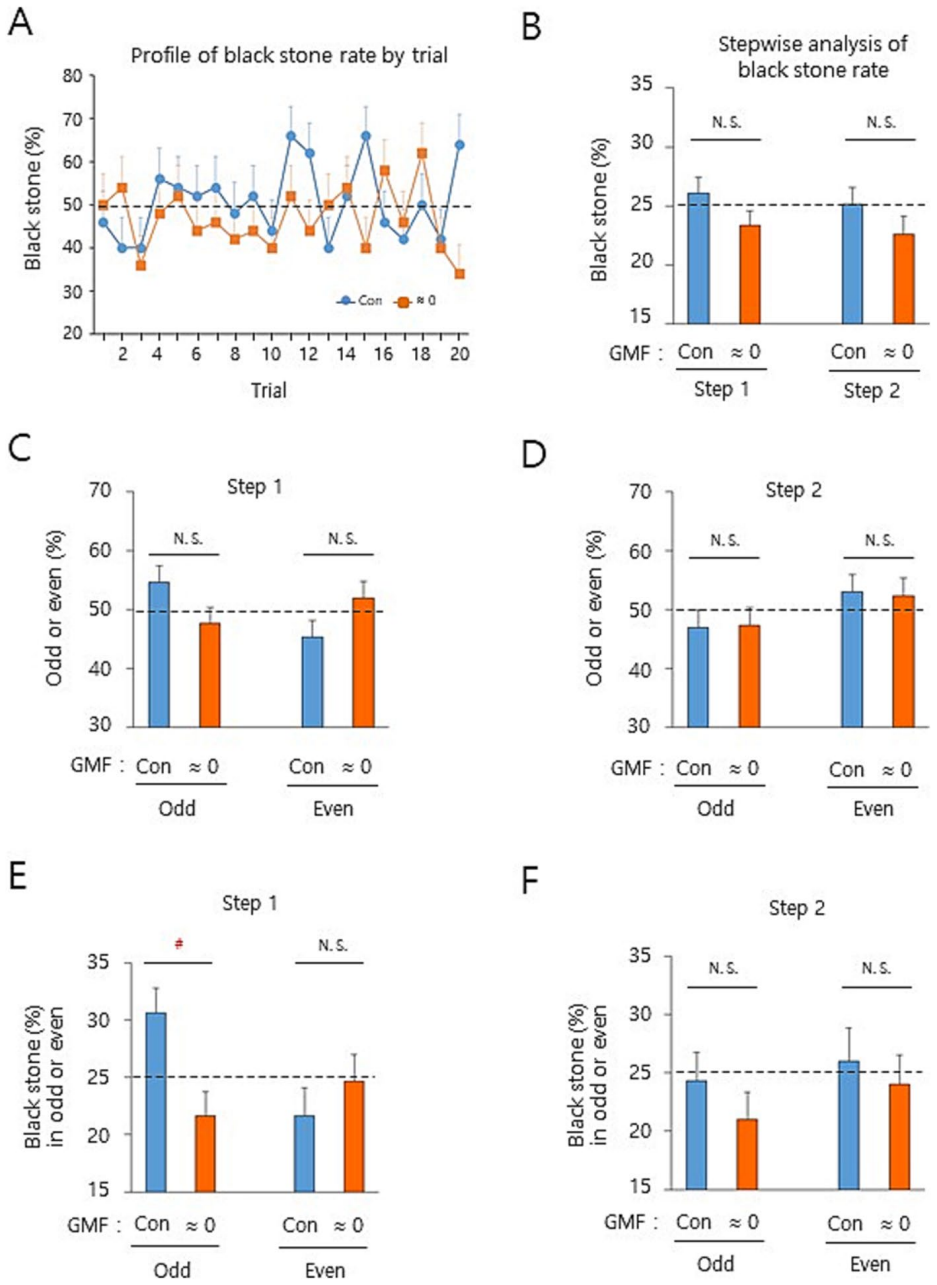
The unconscious decision-making was more likely affected by the geomagnetic field. **(A)** A profile of the black stone (%) for the north seat players was displayed by a time series analysis. The same data in Figure 2B from men were analyzed by trial. Note a continued reduction of the rate by the near-zero GMF, compared to the control, up to the 12th trial with a lag at the first two trials. **(B–F)** The different rates of the north seat players by a stepwise analysis. The data of 1–12th trials were analyzed for the black stone (%) **(B)**, odd or even rate in step 1 **(C)** and step 2 **(D)**, or black stone (%) in odd or even by step 1 **(E)** & 2 **(F)** (see Methods). Note the significant reduction of the black stone (%) by the near-zero GMF in the odd cases of the step 1 **(E)**. GMF, geomagnetic field; Con, control (the ambient GMF); ≈ 0, near-zero GMF; N.S., not significant and ^#^, *p* < 0.025, by the percentile bootstrap analysis; horizontal dashed lines, 25% or 50% theoretical probability for each y-axis index of the graphs; error bars, SEM. Subjects for each Con or ≈ 0 group in the panels **(A–F)** are based on *n* = 50.

To identify the influencing target of GMF in the binary decision-making, we conducted a systematic data analyses from male subjects after ~20 h fast under the opaque screen condition (Figure 2B, Paradigm 2 above). Under these conditions, potential influences including visual or auditory cues from the opponent and surroundings were minimized. The effect of near-zero GMF was assessed in a stepwise manner for black stone (%), compared to controls. First, a time series analysis in the black stone (%) of the north seat players by trial showed that there was a tendency of reduction from the near-zero GMF in the rate up to the 12th trial compared to controls, despite a lag in the first two trials and random patterns at the 13th and 16–18th trial (Figure 3A and Supplementary Data Sheet S11). A stepwise analysis showed that the rate tends to decrease by the near-zero GMF in step 1 and 2 (−10.3% for each step compared to the corresponding control), supporting that the GMF can be a contributing factor for the decision-making in both steps (Figure 3B and Supplementary Data Sheet S11). Although it is considered to be the null hypothesis in the process of stone selection in Supplementary Video S1 [see Figure 1 in Kim et al. (2025)], we attempted to understand how the GMF affected the step 1 and 2 mechanistically. Even though it is not statistically significant, the odd or even rate [odd or even divided by total (%)] of white stones under the control condition showed a tendency of being reversed by the near-zero GMF in the step 1 (Figure 3C and Supplementary Data Sheet S11), whereas the odd or even rate of black stones in the step 2 was not changed by the same treatment (Figure 3D and Supplementary Data Sheet S11). Notably, the subjects answered in the post-experiment questionnaire that they were not able to intentionally grab white stones to be odd or even in the step 1 (approximately 99%), whereas they could intentionally choose one or two black stone(s) in the step 2. Strikingly, the black stone (%) in the odd cases was significantly diminished by the near-zero GMF in the step 1 compared to the control, but not in the step 2 (Figures 3E,F, and Supplementary Data Sheet S11). In contrast, the changes by the near-zero GMF in the even cases of the step 1 and 2 were not significantly detected (Figures 3E,F, and Supplementary Data Sheet S11), suggesting that the GMF was mostly influential in the odd cases of the step 1 to produce the discrepancy between the subconscious decision (step 1) and the conscious decision (step 2). These results suggest that the GMF more likely affects the subconscious binary decision-making (step 1 in this process) than the conscious decision that is intentionally controllable (step 2).

### Magnetic field resonance-dependent mechanism influences decision-making

**Figure 4:**
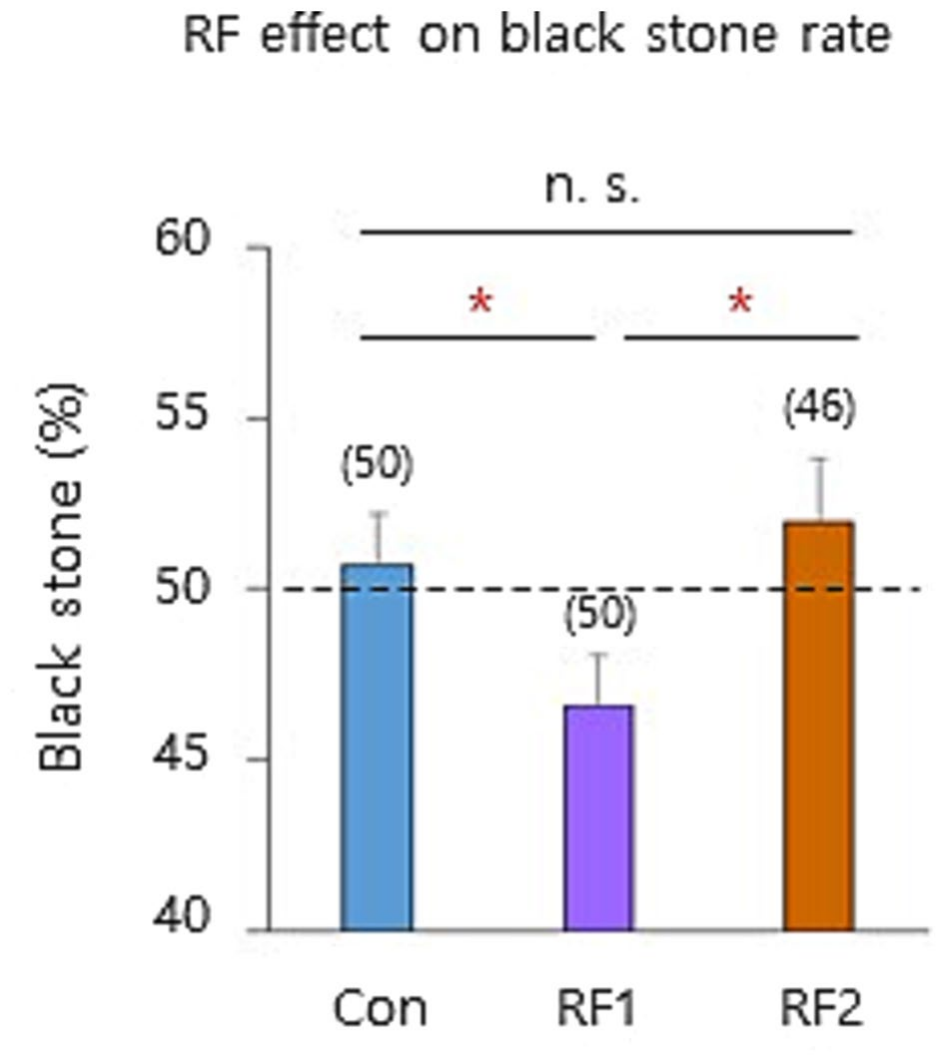
The Larmor frequency magnetic field disrupts the probabilistic decision-making. A significant decrease in the black stone rate by the Larmor resonance frequency 1 (RF 1: 1.260 MHz) was detected compared to the Con, but not by RF 2 (1.890 MHz). Con, control (the ambient GMF); n.s., not significant and *, *p* < 0.05 by a two-sample *t*-test.

**Figure 5:**
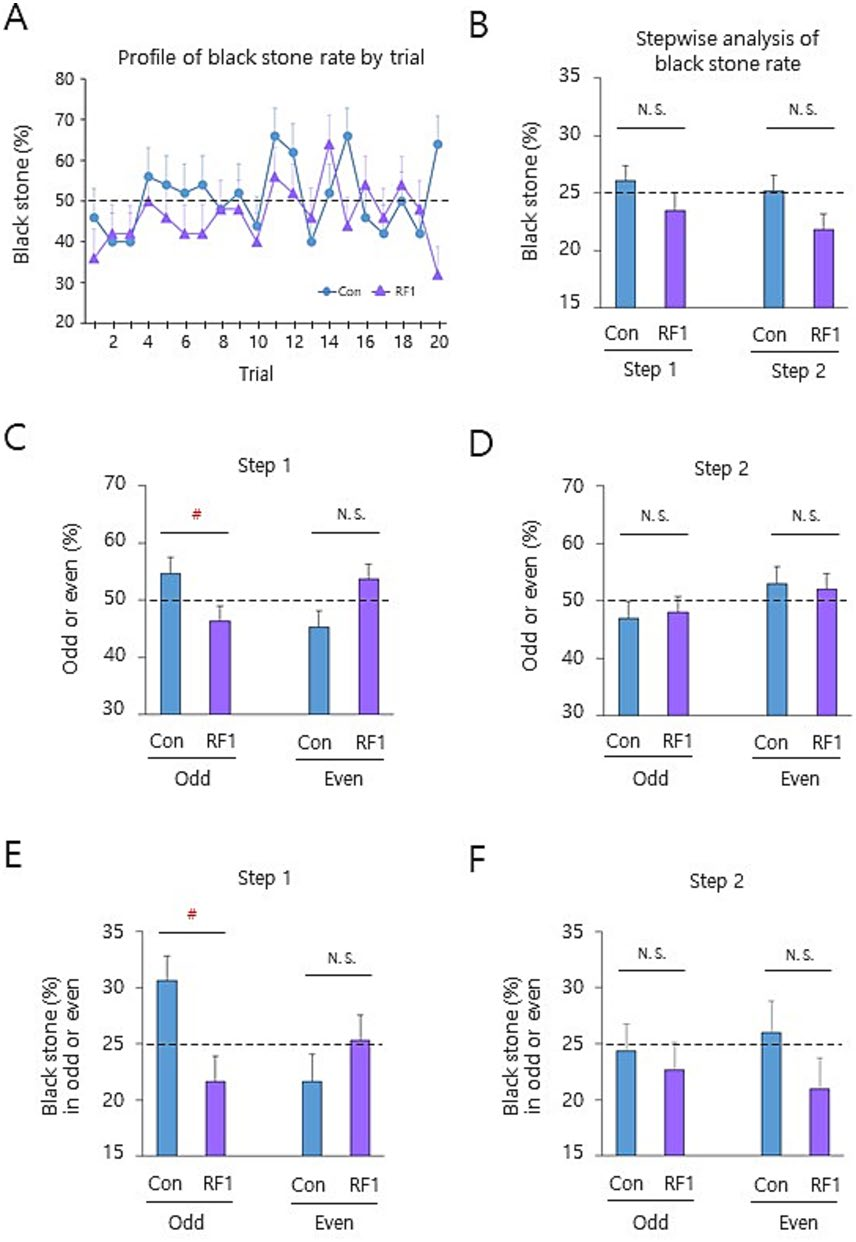
A potential magnetic field resonance mechanism underlying the magnetic sense-dependent decision-making. **(A)** A profile of the black stone (%) of the north seat players in a trial-based analysis. The same data from the RF1 condition in Figure 4 was analyzed by trials. Note a continued reduction of the rate by the RF1, compared to the control, up to the 12th trial with a lag at the second and third trials similar to Figure 3A. **(B–F)** The different rates of the north seat players by a stepwise analysis. The data of 1–12th trials were analyzed for the black stone (%), odd or even rate or black stone (%) in odd or even by steps. Each of the distinct changes by the RF1 in **(B–F)** contributed to the remarkable results of the profile **(A)** and the RF1 condition in Figure 4. Con, control (the ambient GMF); RF1, the 1.260 MHz resonance frequency; N.S., not significant; ^#^, *p*-value <0.025 in odd case **(C)**, > 0.975 in even case **(C)**, and < 0.025 **(E)** by the percentile bootstrap analysis; horizontal dashed lines, 25% or 50% theoretical probability for each y-axis index of the graphs; error bars, SEM. Subjects for each Con or RF1 group in the panels **(A–F)** are consistently *n* = 50.

To investigate the underlying mechanism of GMF-modulated decision-making, the same experimental conditions were employed as Figure 3. Since our previous studies suggested that the blue-light activated radical pair (Hore and Mouritsen, 2016) may underlie human magnetic sense (Chae et al., 2019; Chae et al., 2022), we investigated whether the magnetic field resonance-dependent magnetoreception (Chae et al., 2022) is involved in the decision-making. Particularly, the electron Larmor frequency (Hore and Mouritsen, 2016) (1.260 MHz, radiofrequency 1: RF1) that disrupts GMF sensing, was provided vertically (37° relative to the ambient GMF) to the north seat players (Chae et al., 2022) (Paradigm 3 above). The black stone (%) was significantly reduced by the frequency (RF1; *t*(98) = 1.97, *p* < 0.05), but not by the non-resonance frequency (1.890 MHz, RF2; *t*(94) = − 0.53, *p* > 0.05) (Hore and Mouritsen, 2016; Chae et al., 2022), indicating that a magnetic field resonance-dependent mechanism mediated the GMF-modulated decision-making (Figure 4 and Supplementary Data Sheet S12). The same time series and stepwise analyses performed in Figure 3 revealed that the RF1 specifically disrupted the GMF-modulated decision-making in a very similar way as the near-zero GMF (Figure 5 and Supplementary Data Sheet S13), confirming that GMF sensing notably affected the subconscious decision-making (step 1), and more effectively influenced the odd cases of the step.

## Discussion

In this study, we assessed whether the GMF plays a role in affecting our subconscious decision-making including stone selections in Go games. Our results strongly suggest that the implication of GMF is noticeable in probabilistic *abstract* decision-making, when there is insufficient information for making a decision (Stevens and Ruxton, 2018; Bogacz et al., 2006; Kilpatrick et al., 2019). In our post-experiment questionnaire following stone selection games, the subjects answered that they did not perceive magnetic fields or experience any strange feelings during the game (approximately 98%). The subconscious GMF-modulated decision-making framework may give us a seminal advantage over the ordinary sensory modalities (e.g., the five senses). It is noteworthy to consider that some potential confounders, e.g., visual cues from experimenters and surrounding experimental paraphernalia such as the Helmholtz coils, but not the opponent, might have affected the results. Although it would be difficult to completely exclude such visual cues to influence the subconscious decision-making for enhancing the odds of black stone selection, we have observed that it was affected by the cancelation of the ambient GMF, fasting, and the resonance frequency magnetic field. The potential contribution by the visual cues to the discrepancy between theoretical and empirical probabilities was probably overridden by the randomization and counterbalance of other experimental conditions. Indeed, besides the experimental results in the laboratory, the large-scale empirical data from the *bona fide* professional Go matches and *in situ* stone selection games (see Kim et al., 2025) support this possibility, wherein the players carried out the matches or games under random feeding and the ambient geomagnetic conditions including magnetic intensities at the locations.

The present study may suggest that the GMF sensing-dependent probabilistic decision-making in humans might have been a hitherto unknown cause of apparent discrepancies between theoretical and empirical probabilities, which we often encounter. According to the proposed radical pair mechanism for human magnetoreception, an incidence of external magnetic field, e.g., GMF onto the radical pairs formed by ultraviolet-A/blue light in the eyes (Chae et al., 2019; Chae et al., 2022) can change the quantum singlet-triplet ratio of radical pairs (Hore and Mouritsen, 2016). In addition, if a radiofrequency (RF) magnetic field of Larmor frequency is applied to the condition (Chae et al., 2022), this magnetic field can resonate with the magnetic moment of radical pairs to disturb the singlet-triplet ratio, by which aberrant downstream chemical reactions would be produced and subsequently geomagnetic sensing would be interrupted (Hore and Mouritsen, 2016). Human decision-making is primarily involved in neural circuits in the brain regions, such as cerebral cortex, basal ganglia, and thalamus, and particularly the basal ganglia play pivotal roles in adaptive behaviors following reward and punishment (Nonomura et al., 2018). However, there is no report for the potential implication of magnetosensory information in non-spatial decision-making or from retinal cryptochromes, while a recent report suggested a tentative functional link between brain cryptochromes and navigation, possibly through circadian modulation (Xu S. et al., 2021). In fruit flies, cryptochromes expressed in photoreceptor cells interacted with opsins to exert acute behavioral avoidance responses to UV light (Baik et al., 2017), and cryptochromes in the Johnston’s organ of the antennae mediated the GMF-responsive positive geotactic behaviors to modulate static vertical positioning (Bae et al., 2016). Given the plethora of downstream signaling molecules of cryptochromes that were identified in avian retina (Wu et al., 2020) and the proposed molecular interaction models based on experimental data or *in silico* analysis (DeOliveira and Crane, 2024), it is conceivable that unknown visual signals from radical pairs in retinal cryptochromes may influence the neural circuits involved in subconscious decision-making. According to the increasing body of studies on electromagnetic hypersensitivity (EHS) in humans, it cannot be ruled out that the decision-making can also be disrupted by other non-resonant radiofrequencies of environmental electromagnetic fields (EMFs), which were not tested in the present study (Henshawa and Philips, 2025). This possibility could be partly supported by the positive reports (2%) by the subjects, e.g., bizarre feelings, tinnitus, or prickling in the post-experiment questionnaire above, implying that some of the responses might be EHS symptoms by the cancelation of GMF or the applied radiofrequencies that influenced on the brain.

The significant decreases in black stone selection rate by the near-zero GMF and the resonance frequency magnetic field support that ordinary GMF sensing was necessary for equal empirical probability of both players. As a paradox against the theoretically even chance proposed by the quantum phenomenon (Adler, 2014; Albrecht and Phillips, 2014), the magnetic sense-dependent binary decision making in the present study implies that the GMF-mediated modulation might be a channel for affecting subconscious decision-making as an information flow from the quantum to the classical world, manifesting theoretical probability into empirical probability. Nevertheless, the reason why the odd cases but not even cases in the step 1 were the primary influential target in the binary decision-making remains to be obscure. Noticeably, there were somewhat different selection percentages between odd and even stone(s) in the control group in Figure 3C. Based on the previous studies, it is conceivable that the reason could be a subconscious bias of the players who grabbed white stones in which they subconsciously tried taking advantage of odd numbers in white stones by decreasing the judgment accuracy of the opponents in the second step of stone selections. This scenario is supported by the linguistic markedness hypothesis that odd digits are processed more inaccurately than even digits in a variety of different tasks and types of judgments in both men and women (Hines, 1990; Heubner et al., 2018). It is likely that the same stepwise analysis on the women’s data in Figure 2C may produce a similar result as shown in Figure 3C for men. It would be worth comparatively examining the hypothesis and other alternative possibilities in forthcoming studies.

In addition to the previous reports on men’s magnetic responses to the GMF (Chae et al., 2019; Chae et al., 2022), this study demonstrates for the first time that both men and women can sense the GMF to present magnetic behavioral responses. Intriguingly, men and women were influenced by the GMF in decision-making at the same restricted blood glucose level (mean, 5.1 mol/L), despite the subjects’ blood glucose level varied within the normoglycemia conditions (between 4.7 and 5.3 mol/L) by the different fasting durations (Goyal et al., 2009). Given the positive correlation between blood glucose level and generated O_2_^•-^ concentration in the rat retina (Du et al., 2003), this result supports the FADH^•^/ O_2_^•-^ radical scavenging system in the radical pair hypothesis, which emphasizes the crucial role of O_2_^•-^ in magnetoreception at the optimal range of concentration (Kattnig, 2017; Chae et al., 2022). This tentative explanation raises a possibility of the enhanced sensitivity of magnetic field in the cryptochrome-based radical pair in the eyes (Kattnig, 2017; Player and Hore, 2019; Deviers et al., 2022) at the restricted blood glucose level. Interestingly, birds can efficiently reduce the level of reactive oxygen species during migration through the endogenous antioxidant mechanisms (Gutiérrez et al., 2019), even though they normally maintain 2 to 4 folds higher blood glucose level compared to mammals in equivalent body mass (Braun and Sweazea, 2008). Therefore, the optimum level of O_2_^•-^ might be more critical than blood glucose level for magnetoreception, which can be different depending on the species due to subtle differences in the sensing moiety of putative magnetoreceptor molecules (Zoltowski et al., 2019). These possibilities can be tested using *in vitro* human cell systems; for example, the levels of magnetic field-dependent cellular autofluorescence (Ikeya and Woodward, 2021) can be measured at different concentrations of glucose and O_2_^•-^. In the magnetoreceptive organs of animals including migratory birds, *in situ* measurement of those levels can also be conducted for understanding the correlation between glucose and O_2_^•-^.

Alternatively, the near-zero GMF induced alteration of subjects’ circadian clocks might have affected the black stone rate to be significantly lower than the control. This scenario is based on the simulation of circadian period using spin dynamics that predicted the circadian period to be shortened by the magnetic field with lower intensity compared to the average GMF intensity (50 μT) (Zadeh-Haghighi and Simo, 2022). Moreover, the aberration of circadian period may be amplified under the fasting condition as displayed in the present study, depending on the fasting duration, because food is one of the principal zeitgebers modulating factors for human circadian rhythm (Lewis et al., 2020). A growing body of evidence indicates that normal brain activities including cognitive function and navigation can be depressed under the aberrant circadian rhythm (Gudden et al., 2021; Xu S. et al., 2021; Drunen and Eckel-Mahan, 2023). The feasibility of this scenario may be evaluated by examining the gene expressions of biological clock genes in the blood of subjects (Boivin et al., 2003; Schrader et al., 2024) and/or *in silico* simulation of the circadian period (Zadeh-Haghighi and Simo, 2022) under the concomitant conditions of fasting and near-zero GMF in forthcoming studies. Of course, the two scenarios together can be plausible to be implicated in the magnetoresponsive stone selections.

Further, it can be considered that short-term fasting effects on psychological health as an additional physiological pathway involved in the decision-making. A body of studies revealed that the effects of short-term fasting tend to diverge into positive or negative outcomes in the view of emotions depending on the extent of self-emotional control, religious beliefs, previous fasting experience of the subjects, and the difference of assessment tools (Wang and Wu, 2022). In contrast, some recent studies showed that short-term fasting improved olfactory sensitivity to non-food odors and facilitated momentary attention to stimuli from internal organs through alterations in autonomic nervous system function (Schwerdtfeger and Rominger, 2024). Thus, it could be plausible to think that magnetic sensitivity of humans could be enhanced under the short-term fasting conducted in the present study, which might stimulate the magnetic sense-dependent decision-making, although the underlying physiological pathways remain to be elucidated. To understand the possible physiological mechanisms underlying near-zero GMF’s impact on decision-making, we can speculate whether or not decision-making may be impaired in outer space, where the GMF is substantially mitigated. According to the accumulated evidence from *in vitro* and *in vivo* experiments, the absence of GMF in deep space still likely influences a variety of brain activities including cognitive functions, such as memory and decision-makings to a variable extent, depending on the complex contexts, including hypo-gravity and various EMFs from the spacecrafts, etc. (Hart, 2023; Kong et al., 2024). Although the impact of near-zero GMF in deep space can not be easily estimated, an impairment of decision-making could be relatively transient in analogous to the result from the time series analysis on the black stone (%) (Figure 3A) due to the efficient adaptation to the hypo-GMF condition or the impairment can be sustained to an undefined extent if the homeostasis of a person is shifted into a new level under the prolonged period of hypo-GMF circumstances.

Lastly, the present study was conducted based on the hypothesis of radical pair mechanism which is possibly mediated by light-activated cryptochromes in the retina. Particularly, the significant mitigation of the decision-making by the resonant radiofrequency magnetic field strongly supports that the radical pair may play a pivotal role in the non-spatial abstract decision-making. In a broad view, however, it would be needed to consider the possibility that the proposed light-independent magnetosensory pathways (Shibata et al., 2024) also can be engaged in the decision-making. If this is the case, magnetic information could be sensed by an electromagnetic induction rather than by magnetic particles-based torque, and transmitted via the trigeminal or vestibular nerves linked to hindbrain pathways (Johnsen and Lohmann, 2005; Shibata et al., 2024). Following studies are sought to shed light on an avenue for arguments about the contrasting hypotheses.

Overall, the findings from the present study may provide unique insights into the function of magnetic sensing in 3-dimensional spaces and the newly identified mechanistic entity to manifest the theoretical probability in the real world. The potential principle and mechanism proposed here may actually impose on other binary decision-makings including coin toss or penalty kick direction in soccer. The potential discrepancy between theoretical and empirical probability from these events in the past or future could be assessed using enormous empirical data. This present study could be a corner-stone for identifying mysterious entities in influencing subconscious decision-making, e.g., GMF. The ever-existing GMF may not only converge empirical probability into theoretical probability in most cases, but also ironically can produce unequal empirical probability under particular magnetic conditions in some practical decision-making cases. Notably, our findings are likely raising more questions than providing answers for elucidating the role of GMF in subconscious decision-making. However, we would like to address unique perspectives for understanding the discrepancies between theoretical and empirical probability, and the following relentless efforts will contribute to reveal the mechanisms of GMF in influencing numerous subconscious decision-makings.

## Data Availability

The original contributions presented in the study are included in the article/Supplementary material, further inquiries can be directed to the corresponding author.
