# Corrigendum: Magnetic sense-dependent probabilistic decision-making in humans

**DOI:** 10.3389/fnins.2025.1591645

**Published:** 2025-03-25

**Authors:** In-Taek Oh, Soo-Chan Kim, Yongkuk Kim, Yong-Hwan Kim, Kwon-Seok Chae

**Affiliations:** ^1^Brain Science and Engineering Institute, Kyungpook National University, Daegu, Republic of Korea; ^2^Department of Electrical and Electronic Engineering, Research Center for Applied Human Sciences, Hankyong National University, Anseong, Republic of Korea; ^3^Department of Mathematics, Kyungpook National University, Daegu, Republic of Korea; ^4^Neuroscience Program, School of Allied Health Sciences, Boise State University, Boise, ID, United States; ^5^Department of Biology Education, Kyungpook National University, Daegu, Republic of Korea

**Keywords:** decision-making, probability, magnetic sense, humans, binary choice, geomagnetic field, magnetoreception, magnetic field resonance

In the published article a reference was cited incorrectly. A correction has been made to the **Introduction**, Paragraph 4. This sentence previously stated:

“To test this hypothesis, we adopted the zero-sum stone selection of Go games (Kim et al., 2025) to investigate the potential implication of GMF for inducing a discrepancy between the empirical and theoretical probability.”

The corrected sentence appears below:

“To test this hypothesis, we adopted the zero-sum stone selection of Go games (Chae et al., 2023) to investigate the potential implication of GMF for inducing a discrepancy between the empirical and theoretical probability.”

The authors apologize for this error and state that this does not change the scientific conclusions of the article in any way. The original article has been updated.

