# Supplementary material for: Magnetic sense-dependent probabilistic decision-making in humans
Source: Front Neurosci. 2025 Mar 7;19:1497021. doi: 10.3389/fnins.2025.1497021 (PMC11925921; doi:10.3389/fnins.2025.1497021)
Supplement: Supplementary file 1 [file Data_Sheet_1.pdf]

Record form (Stone selection game)

* Date : . . Hr : Min : * Location (room) :

* Player 1 (A) : Gender : Player 2 (B) : Gender : Game : Set :

Trial	Process		Result
	Step 1 (number of white stones)	Step 2 (number of black stones)	The player gets black stone
(Example 1)	A 17	B 1	B
(Example 2)	B 14	A 2	A
(Example 3)	A 15	B 2	A
1	A	B	
2	B	A	
3	A	B	
4	B	A	
5	A	B	
6	B	A	
7	A	B	
8	B	A	
9	A	B	
10	B	A	
11	A	B	
12	B	A	
13	A	B	
14	B	A	
15	A	B	
16	B	A	
17	A	B	
18	B	A	
19	A	B	
20	B	A	

Black stone rate (Set)
A: % (/20)
B: % (/20)

Black stone rate (Game)
A: % (/40)
B: % (/40)

Record form (Stone choice)

* Date : 2022. 8 .29 Hr : Min 16 : 48 * Location (room) : 102

* Player 1 (A) : [redacted] Gender : M Player 2 (B) : [redacted] Gender : M Game : 1 Set : 2

Trial	Process		Result
	Step 1 (number of white stones)	Step 2 (number of black stones)	The player gets black stone
(Example 1)	A 17	B 1	B
(Example 2)	B 14	A 2	A
(Example 3)	A 15	B 2	A
1	A 17	B 2	A
2	B 16	A 2	A
3	A 20	B 1	A
4	B 16	A 2	A
5	A 14	B 2	B
6	B 14	A 2	A
7	A 14	B 2	B
8	B 16	A 2	A
9	A 17	B 1	B
10	B 15	A 2	B
11	A 16	B 2	B
12	B 16	A 2	A
13	A 15	B 2	A
14	B 16	A 2	A
15	A 19	B 2	A
16	B 15	A 1	A
17	A 17	B 1	B
18	B 15	A 2	B
19	A 15	B 1	B
20	B 18	A 2	A

Black stone rate (Set)
A: 60 % (12/20)
B: 40 % (8/20)

Black stone rate (Game)
A: 57.5 % (23/40)
B: 42.5 % (17/40)
