# Supplementary material for: Magnetic sense-dependent probabilistic decision-making in humans
Source: Front Neurosci. 2025 Mar 7;19:1497021. doi: 10.3389/fnins.2025.1497021 (PMC11925921; doi:10.3389/fnins.2025.1497021)
Supplement: Supplementary file 2 [file Data_Sheet_2.pdf]

Calculation formulas for the time series and stepwise analyses

For the stepwise analysis in Figs. 3 and 5, the raw data of the 1–12th trials in those figures were applied to the calculation formula below. 1) For ‘black stone (%)’ of a subject in the step 1, the number of trials with success for black stone in the step 1/total number of trials for a subject (*i.e.*, 12) × 100; 2) For ‘black stone (%)’ of a subject in the step 2, the number of trials with success for black stone in the step 2/total number of trials for a subject (*i.e.*, 12) × 100; 3) For ‘odd/even (%)’ of a subject in the step 1, the number of trials with odd/even white stones/total number of trials for grabbing white stones in the step 1 (*i.e.*, 6) × 100; 4) For ‘odd/even (%)’ of a subject in the step 2, the number of trials with one or two black stone(s)/total number of trials for grabbing black stone(s) in the step 2 (*i.e.*, 6) × 100; 5) For ‘black stone (%) in odd/even’ of a subject in the step 1, the number of trials with success for black stone in the case of odd or even white stones/total number of trials for grabbing white stones in the step 1 (*i.e.*, 6) × 100; 6) For ‘black stone (%) in odd/even’ of a subject in the step 2, the number of trials with success for black stone in the case of one or two black stone(s)/total number of trials for grabbing black stone(s) in the step 2 (*i.e.*, 6) × 100. For each of the group rates in the analyses above, the rates of all the subjects were averaged.
