# Supplementary material for: Magnetic sense-dependent probabilistic decision-making in humans
Source: Front Neurosci. 2025 Mar 7;19:1497021. doi: 10.3389/fnins.2025.1497021 (PMC11925921; doi:10.3389/fnins.2025.1497021)
Supplement: Supplementary file 14 [file Table_1.pdf]

Table S1. Magnetic field parameters for modulated GMF experiments.

MF	Player seat [a]	Components (μT)			Total Intensity (μT)	Relative total Intensity (%) [b]
		X	*Y*	*Z*		
Con	N	30.7	− 6.6	32.4	45.1	100
	S	29.3	− 4.1	33.2	44.5	99
≈ 0	N	0.0	0.0	0.0	0.0	0 [c]
	S	17.8	− 5.8	34.4	39.2	88 [d]

The intensities of the magnetic fields were the means from measurements on the glabella of the three subjects with the highest, median, and the lowest sitting height on the chair. [a] The north seat (N) and south seat (S) are located at the center and outside of the Helmholtz coils, respectively, on the geomagnetic north-south axis. [b] The relative total intensity of the S-seat magnetic field was compared to that of the corresponding N-seat in each GMF condition. [c, d] Relative total intensity at the N and S seats in the ≈ 0 condition was compared with the total intensity at the N and S seats in the Con condition, respectively. MF, magnetic field. MF conditions were named with respect to the N seat; Con, the ambient GMF; ≈ 0, near-zero GMF.
